# A Yoga and Compassion Meditation Program Reduces Stress in Familial Caregivers of Alzheimer's Disease Patients

**DOI:** 10.1155/2013/513149

**Published:** 2013-04-18

**Authors:** M. A. D. Danucalov, E. H. Kozasa, K. T. Ribas, J. C. F. Galduróz, M. C. Garcia, I. T. N. Verreschi, K. C. Oliveira, L. Romani de Oliveira, J. R. Leite

**Affiliations:** ^1^Department of Psychobiology, Universidade Federal de São Paulo-UNIFESP, 925, Rua Napoleão de Barros, 04024-002 São Paulo, SP, Brazil; ^2^Departiment of Research, Instituto Appana Mind de Desenvolvimento Humano, 05436-020 São Paulo, SP, Brazil; ^3^Instituto do Cérebro, Hospital Israelita Albert Einstein, 05601-901 São Paulo, SP, Brazil; ^4^Department of Medicine, Division of Endocrinology, Universidade Federal de São Paulo-UNIFESP, 04023-062 São Paulo, SP, Brazil

## Abstract

Familial caregivers of patients with Alzheimer's disease exhibit reduced quality of life and increased stress levels. The aim of this study was to investigate the effects of an 8-week yoga and compassion meditation program on the perceived stress, anxiety, depression, and salivary cortisol levels in familial caregivers. A total of 46 volunteers were randomly assigned to participate in a stress-reduction program for a 2-month period (*yoga and compassion meditation program*—YCMP group) (*n* = 25) or an untreated group for the same period of time (control group) (*n* = 21). The levels of stress, anxiety, depression, and morning salivary cortisol of the participants were measured before and after intervention. The groups were initially homogeneous; however, after intervention, the groups diverged significantly. The YCMP group exhibited a reduction of the stress (*P* < 0.05), anxiety (*P* < 0.000001), and depression (*P* < 0.00001) levels, as well as a reduction in the concentration of salivary cortisol (*P* < 0.05). Our study suggests that an 8-week yoga and compassion meditation program may offer an effective intervention for reducing perceived stress, anxiety, depression, and salivary cortisol in familial caregivers.

## 1. Introduction

The aging of the worldwide population has contributed to the increasing incidence of dementia-causing chronic degenerative diseases, notably Alzheimer's disease [1]. Alzheimer's disease is the most common form of dementia and is characterized as a progressive brain disorder with loss of reasoning, memory, language skills, and the ability to maintain an independent life [2]. The caregivers of Alzheimer's disease patients are typically family members [3]. The physical and mental burden imposed on caregivers frequently results in poor quality of life [4]. The physical, mental, social, and financial burden to which caregivers are exposed to might result in an increased risk of acute myocardial infarction and death [5–7]. Among the several psychosocial contexts experienced by caregivers, the following are particularly noteworthy: stress, social exclusion, depression, emotional isolation, burnout of personal relationships, loss of life perspectives, sleep disorders, and abuse of psychotropic substances [8–13]. Several studies of Alzheimer's disease patients and caregivers stress the importance of the medical team orienting the familial caregivers beginning in the earliest stages of disease, and plans for such support interventions have been suggested in the literature [14–16]. Yoga and meditation are among the modalities of intervention that might be applied to this population.

 One of the most appropriate definitions of yoga is found in documents dated approximately 2,000 years ago, namely, Patanjali's Yoga sutras. One of such sutras defines yoga as follows: yoga is the restriction of the fluctuations of mind-stuff [17]. Thus, one of the aims of yoga is the development of mindfulness; several meditation techniques exhibit similar goals. Although meditation is a component of yoga, there are other approaches to meditation, such as compassion meditation, which originated in Buddhism, that are not associated with bodily practices, as in yoga. For this reason, both terms, yoga and meditation, will be used throughout the present paper. 

Practices related with yoga and meditation have been investigated as potential techniques for reducing stress [18–21], anxiety [21–23], and depression [21, 23]; for improving physical and mental wellbeing and several domains of cognition [23, 24]; and for enhancing quality of life [25, 26]. During these practices, the participant develops the ability of focused attention to the present moment, instead of being arrested by thoughts and ruminations [18]. This mental stability seems to be related to sympathetic reduction and conversely improvement in the parasympathetic activity contributing to stress and anxiety reductions [18–20, 27]. 

Few studies investigated the stress levels of the caregivers of patients with chronic diseases before and after such interventions as yoga and meditation. A pilot study applied a program known as Inner Resources as therapy to 12 women responsible for the care of relatives with dementia. The protocol developed by that program included meditation, yoga body positions and respiration techniques, and visualizations and repetitions of specific sounds, which are known as mantras. The application of specific questionnaires allowed the researchers to detect improvement in the caregivers' state of depression and anxiety [22]. A more recent study found similar results upon assessing the effects of meditation on perceived stress and related indices of psychological status in Alzheimer's disease patients and their caregivers [27]. However, these results must be interpreted cautiously, as neither study included a control group, and the sample sizes were small in both cases. 

 In addition to the emotional stress that caregivers suffer upon seeing their relative deteriorate physically and mentally, caregivers of patients with Alzheimer's disease must perform physical labor. Therefore, the aim of the present study was to investigate whether the practice of a yoga and compassion meditation program might alter the stress, anxiety, and depression levels of familial caregivers of patients with Alzheimer's disease, as measured on standardized questionnaires. The present study also sought to assess whether the intervention modified the caregivers' morning cortisol level.

## 2. Methods

### 2.1. Study Participants

The present study was approved by the Research Ethics Committee of UNIFESP, Protocol no. 1528/07. The study volunteers were recruited by means of announcements on radios and newspapers placed the Press Consultancy of UNIFESP and were also referred by the Brazil Alzheimer's Association (Associação Brasileira de Alzheimer—ABRAZ). The inclusion criteria were as follows: older than 18 years of age, minimum educational level corresponding to complete elementary education, and exhibiting at least the resistance stage of stress on Lipp's Stress Symptoms Inventory for Adults (LSSI) [28]. The exclusion criteria were as the following: diagnosis of Cushing's syndrome; ongoing treatment with corticoids per any route, including the topical and nasal ones in the previous 30 days; diagnosis of asthma or chronic obstructive pulmonary diseases (COPD); use of alcohol (more than five drinks per week) or drugs of abuse; and regular practice of yoga, meditation, or similar techniques. The use of tranquilizers was not an exclusion criterion, provided that the volunteers started it before the study and did not discontinue it during the study period. A total of 53 familial caregivers of Alzheimer's disease patients were selected. Forty-six of the subjects completed all of the stages of the study after random allocation into 2 groups: 25 volunteers (22 females and 3 males) participated in the yoga and compassion meditation program (YCMP group), and 21 (19 females and two males) volunteers made up the control group (CG), which was a nontreated group. For ethical reasons, after the end of the study, the volunteers in the CG also participated in the suggested intervention. The participants visited the Psychobiology Department of UNIFESP, where they were subjected to clinical examination to assess their overall health, filled in identification questionnaires, and signed a free and informed consent form. Next, the volunteers filled in the psychological scales and inventories and were instructed to the procedures to collect salivary samples to measure cortisol levels. These same assessments were performed two months later in both groups.

### 2.2. Outcome Measures

 The following instruments were used to measure the psychological aspects.

- Lipp's Stress Symptoms Inventory for Adults (LSSI), which has been frequently used in Brazil in recent years and categorizes stress according to a four-phase model, namely, alertness, resistance, quasiexhaustion, and exhaustion according to the amount of physical and psychological stress symptoms [28].
- Beck Depression Inventory (BDI) and Beck Anxiety Inventory (BAI), both comprising 21 questions describing depression and anxiety symptoms to which subjects respond on a four-point scale [29–32].
- In addition, the salivary cortisol level was used as a measure of physiological stress. For that purpose, 8 samples were collected from each participant. Four samples were collected before the intervention: two samples were collected on 2 consecutive days, both immediately and 30 minutes after waking up, under fasting conditions. Additional 4 samples were collected following the same procedures after intervention. Morning was selected as the time for sample collection because it corresponds to the maximum peak of cortisol throughout the day [33–36]. As the morning cortisol level is sensitive to light—the greater the luminosity, the higher the cortisol concentration—[35], the volunteers were oriented to perform the first sample collection immediately upon waking up and in the dark, and the second sample was collected 30 minutes later under exposure to natural light. We analyzed the results based on the average results of the first day samples, the second day samples, and the overall average of both days. The samples were processed at the Laboratory of Steroids of the Department of Endocrinology of UNIFESP. The samples were centrifuged at 3,000 rpm for 5 minutes, and the supernatant was stored at −20°C until testing, which was performed by means of radioimmunoassay (RIA). Each sample was tested in duplicate using 100 *μ*L of supernatant without any previous extraction [37]. An antibody (cortisol-3-(O-carboxymethyl)-oxime-bovine) highly specific for cortisol (100%) but without significant cross-reactivity with other steroids (<1%) was used.

### 2.3. Intervention

 The stress reduction program lasted 2 months, with sessions lasting 1 hour and 15 minutes, 3 times per week. The participants had to attend 1 live session per week, totaling eight live sessions, whereas the other 2 weekly sessions (16 sessions) were performed at home with the aid of a DVD. The program included the following traditional *Hatha Yoga* exercises.

- Yoga body poses (*asanas*) (25 min): *Sukhasana*—practitioner sits with the legs crossed and the back straight. *Vajrasana*—practitioner sits on the heels, keeping the legs and thighs together, and the back straight. *Yoga-Mudra*—practitioner sits on the heels, keeping the legs and thighs together, and the back bent forward. *Paschimottanasana*—practitioner sits with the knees stretched, feet together, and back bent forward. *Ardha-Matsyendrasana*—practitioner sits intertwining the legs, the back straight, and twists the back to look behind; this movement is performed in both directions. *Shavasana*—practitioner lies on the floor, on the back, with the arms along the body, and the knees extended. *Naukasana*—practitioner lies on the floor, on the back, then raises the legs and trunk from the floor, and keeps this position as long as it is comfortable. *Bhujangasana*—practitioner lies on the floor, on the abdomen, with the palms of the hands on the floor, at the pectoral level. Upon the instructor's command, the practitioner raises the trunk gently from the floor while stretching the back. *Ardha*-*Shalabhasana*—practitioner lies on the floor, on the abdomen, with the arms along the body, legs together, and knees stretched. Upon the instructor's command, the practitioner raises one thigh from the floor; this movement is performed on both sides. *Standing Chakrasana*—practitioner stands with the feet together, raises the right arm, and bends the trunk toward the left side; this movement is performed on both sides. *Vrikasana*—practitioner stands with the feet together, removes the right foot from the floor, and places it on the internal side of the left thigh. Next, he/she joins the palms of the hands in front of the chest, raises the arms above the head, and remains in this position, keeping the balance, as long as it is comfortable. This movement is performed on both sides. *Sarvangasana*—at the beginning of this movement, the practitioner lies on the floor, on the back. The aim of this exercise is to raise the legs, thighs, and hips from the floor, that is, to attain an upside-down position, while supporting the body on the upper part of the back, upper arms up to the elbows, and the back of the head. The *asanas' *average duration of each movement was approximately one and a half minutes [38, 39].
- Exercises involving awareness and voluntary regulation of breath (*pranayamas*) (25 min): *Adhama Pranayama*—free, predominantly abdominal respiration. *Bhastrika*—forceful expirations through the nose followed by passive inspirations. *Ujjayi*—slow and complete inspiration, retention of the air in the lungs, and slow expiration through partially closed glottis. *Surya Bhedana*—inspiration through the right nostril followed by expiration through the left nostril. *Chandra bhedana—*inspiration through the left nostril followed by expiration through the right nostril.* Nadi Shodhana*—inspiration through the left nostril, retention of air in the lungs, expiration through the right nostril, inspiration through the right nostril, retention of air in the lungs, and expiration through the left nostril. *Kapalbhati*—forceful, short, and fast expiration through the nostrils followed by passive inspiration [38, 39]. The pranayamas' average duration of each one was approximately three minutes [40, 41] (12.5 min).
- Meditational practices: mindfulness meditation adapted for Western psychology, originating in Buddhist meditation, involves paying sustained attention to sensations, perceptions, and thoughts with suspension of judgment [18].
- Compassion meditation: compassion meditation (*karuna*) emphasizes the voluntary production of compassionate feelings for all living beings based on the assumption that all beings aspire to happiness and seek to release themselves from suffering and its causes [42, 43] (12.5 min).

### 2.4. Statistical Analysis

To establish whether the groups were similar before the intervention, the chi-square (*χ* ^2^) test was applied to the nominal and ordinal qualitative variables, and Student's *t*-test was applied to the quantitative variables. To analyze the psychological (BDI and BAI) and physiological (cortisol level) measurements, 2-way ANOVA was applied (YCMP and CG groups) (before and after intervention) followed by Tukey's test when necessary. The differences between the groups and the time-points were investigated according to their statistical significance and as was the interaction between these factors. With respect to LSSI, the chi-square test (*χ* ^2^) was used to compare the groups before and after intervention. To assess the effect of intervention, that is, to compare the results after intervention to the ones before intervention, McNemar's test was applied to each category of phases of stress. All of the statistical analyses were performed using software Statistica version 10.1, and the significance level was established as *P* ≤ 0.05.

## 3. Results

Before the intervention, both groups were statistically homogeneous with respect to the following variables: male gender (YCMP, *n* = 3, 12%) (CG, *n* = 2, 10%) and female gender (YCMP, *n* = 22, 88%) (CG, *n* = 19, 90%); educational level, complete elementary education (YCMP, *n* = 1, 4%) (CG, *n* = 0, 0%), complete secondary education (YCMP, *n* = 11, 44%) (CG, *n* = 5, 24%), and complete higher education (YCMP, *n* = 13, 52%) (CG, *n* = 16, 76%); family income (minimum wage, MW = BRL 622.00) up to five times the MW (BRL 3,110.00) (YCMP, *n* = 6) (CG, *n* = 7), more than five and up to 10 times the MW (BRL 3,110.00 to BRL 6,220.00) (YCMP, *n* = 11) (CG, *n* = 6), over 10 times the MW (above BRL 6,220.00) (YCMP, *n* = 8), (CG, *n* = 8); age (YCMP, 55.5 ± 8.1 years), (CG, 53.4 ± 8.2 years); and length of caregiving (YCMP, 4.2 ± 3.3 years) (CG, 5.7 ± 3.7 years). 

 The participants in the YCMP group reported 100% commitment to the suggested program. This level of adherence was most likely facilitated by the relative flexibility of the program, as the live sessions could be attended at five different times and at three different locations in the city of São Paulo. Further, the use of the DVD for practice at home facilitated the optimal rate of adherence to the protocol. 

Table 1 shows the frequency of distribution of the phases of stress manifestation in both groups. Before the intervention, the groups were statistically similar in this regard: *χ* ^2^ (*P* > 0.05). No participant in any group was in either the absent or alert stages, 86% in the CG and 80% in the YCMP group were in the phase of resistance, 14% in the CG, and 12% in the YCMP group were in the phase of quasi-exhaustion, and only 2 individuals in the YCMP group were in the phase of exhaustion. However, after intervention, the groups exhibited statistically significant differences: *χ* ^2^ (*P* < 0.05). No participant in the CG group was in either the absent or alert phase, whereas 68% of the participants in the YCMP shifted to the phase characterized by absence of stress manifestations. Table 1 further demonstrates that statistically significant diagnostic changes occurred in the YCMP group. Before the intervention, 20 volunteers were in the phase of resistance, and none of the volunteers was in the phase of absence. After the intervention, only 8 volunteers were in the phase of resistance, and the other 17 volunteers were in the phase of absence of stress: *McNemar*'s test (*P* < 0.05). Conversely, the CG did not exhibit any statistically significant change between the beginning and the end of intervention.

Table 2 demonstrates that the average depression (BDI) and anxiety (BAI) scores exhibited statistically significant interactions with the time point and group factors. These interactions suggest that the groups' profiles differed over time. Both variables exhibited a significant reduction in the YCMP group in addition to a difference between the groups after the intervention. The YCMP group exhibited a 51.2% reduction in the BDI score after the intervention, whereas the CG exhibited an increase of 9.5% at the same time point. The YCMP group exhibited a 49.4% reduction in the BAI score, whereas the CG exhibited an increase of 10% at the same time point. 

Table 3 presents the salivary cortisol concentration values corresponding to the average of the two measurements on the first sampling day (day one), the average of the two measurements on the second sampling day (day two), and the average of the four measurements performed over both days (day one and day two). The YCMP group exhibited a statistically significant reduction in the salivary cortisol level on day two (not in day one), as well as a reduction in the average of day one + day two before and after intervention. The differences, according to the table, are not significant in group factor, but they are significant in time and interaction factors. The control group did not exhibit any significant change. Some samples had an insufficient volume of saliva to perform cortisol analysis and were lost as a result. On the first day of the preintervention phase for the YCMG group, we lost 20% of the saliva samples for cortisol analysis, and on the second day we lost 18%, giving an overall loss of 19%. In the same group, in the post-intervention phase, we lost 30%, 26% and 28% of saliva samples, respectively, for the same reason. There were also losses in the CG: on the first day of the preintervention phase there was a loss of 11.90% of the samples, 14.29% on the second day, giving an overall loss of 13.10%. In the post-intervention phase, we lost 14.29%, 28.57% and 21.43%, respectively.

## 4. Discussion

The present study sought to establish whether an intervention using yoga and compassion meditation exercises could change stress, anxiety, and depression scores and reduce the salivary cortisol levels of familial caregivers of Alzheimer's disease patients. Among the most relevant findings were the changes in the stress manifestation phases of the participants in the YCMP group from the categories of resistance (*N* = 20), quasiexhaustion (*N* = 3), and exhaustion (*N* = 2) to absence (*N* = 17) and resistance (*N* = 8), thereby demonstrating the effectiveness of the intervention. The changes exhibited by the YCMP group in the stress indices measured by LSSI suggest that our program was able to reverse the stress-related symptoms exhibited by that group (Table 1). 

Elevations in the hypothalamic pituitary adrenal (HPA) axis are common occurrences under conditions of chronic psychological stress and result in increased cortisol levels. Such occurrences are associated with hippocampal volume loss and memory impairment and might increase the risk of dementia in older adults [44–46], eventually creating a vicious circle, whereby familial caregivers of patients with Alzheimer's disease become future patients. Kang et al. [47] applied a program similar to the one used in this study and observed a statistically significant reduction in the stress and anxiety levels of 16 nursing students paired with 16 volunteers, who constituted the control group. In the study, the intervention group performed the suggested program, comprising one weekly session, with each session lasting from one hour and a half to two hours over two months. The intervention group exhibited a statistically significant reduction in the stress and anxiety scores, thus indicating that this type of intervention might be useful to reduce stress in the healthcare professionals directly involved with patient care tasks [47]. The literature includes further evidence indicating that the application of these practices might be useful when applied to caregivers [22, 27], and our study corroborates such findings. 

 Our data demonstrate statistically significant reduction in the depressive symptoms among the participants in the YCMP group. An emergent body of evidence points to the possible therapeutic effects of the practice of aerobic or anaerobic exercises on depression [48–50]. Such exercises, meanwhile, might be either mindful or nonmindful physical exercises. A systematic review analyzed possible therapeutic differences between these two types of exercise. The analyzed results were provided by 12 studies that complied with the methodological criteria applied by the authors for inclusion in that review. According to the authors, both mindful and nonmindful exercises proved to be effective in reducing depression symptoms [50]. It is worth noting that among other activities, the volunteers in our study were subjected to the practice of hatha yoga, which includes physical exercise, most of them anaerobic, to achieve that same goal. Nevertheless, our results cannot be discussed on the grounds of the therapeutic effects of the practice of physical exercise alone because our purpose was to subject the volunteers to a program that included mindful exercises and compassion meditation. Therefore, our results must be attributed to the program as such and not merely to one of its parts. In addition, Kozasa et al. investigated the efficiency of a program that included mantra meditation and pranayamas, known as Siddha Samadhi Yoga, in reducing scores of anxiety and depression in participants with anxiety complaints. This program includes meditation exercises associated with exercises of respiration (pranayamas). A total of 22 volunteers participated in that study, with 14 volunteers being allocated to the intervention group and eight to the control group. The volunteers in the intervention group were oriented to practice the exercise protocol every day of the week, three times per day, in 15-minute sessions. At the end of the study, as in ours, the intervention group exhibited a significant reduction in the anxiety and depression scores compared to the control group [51]. 

 A relationship between practices involving meditation and the endocrine and immune systems has been analyzed in the scientific literature. Witek-Janusek et al. observed a reduction in cortisol concentrations and an improvement of immune system function among women diagnosed with breast cancer who were subjected to the mindfulness-based stress reduction (MBSR) program compared to a control group also comprising women with breast cancer [52]. In the present study, we chose to measure the salivary cortisol levels as a physiological variable sensitive to the behavioral modifications to which the volunteers were subjected. As presented in Table 3, our results indicate that the changes exhibited by the YCMP group, with respect to the cortisol levels of the samples collected on the second day and the total average of the samples collected on the first and second days, were higher compared to the control group. Our results indicate that the participants in the YCMP group developed psychophysiological adaptations associated with the participation in the indicated program, although we did not find statistically significant differences between the groups in the measurements of salivary cortisol performed after the intervention. 

 During the study, we hypothesized that the caregivers' menstrual cycle phase might interfere with the results; however, we were not able to control this variable. However, one study investigated the morning salivary cortisol levels in 99 women and did not find any correlation between the menstrual cycle phase and salivary cortisol levels [53]. It is also worth noting that, according to the literature, the earlier individuals wake, the higher the morning cortisol levels are [54, 55]. As a function of the characteristics of our study sample, all of the participants awoke between 7:00 and 8:00 am. Although this fact most likely reduced the probability of interferences related to different waking times, we were not able to control this variable in a more rigorous manner. One issue deserving discussion is the fact that nicotine is known to be a powerful stimulator of the hypothalamic-pituitary-adrenal axis (HPA) [56]. Consequently, smoking could interfere with the correlation between cortisol levels and stress. Nevertheless, only two volunteers (both in the YCMP group) in our total sample (46 familial caregivers) were smokers. We believed that this low number noticeably reduced the possibility of interference associated with smoking. 

Comparing to previous studies with familial caregivers, [22, 27], the present study is randomized and controlled and has a bigger sample. However, the CG had no treatment, and it could be a limitation of the study. We suggest that similar studies include an active control group which can be a stress discussion group, for example. Another interesting data not collected in this study was the impact of the caregiver's stress reduction on the Alzheimer's patients.

Like mentioned in the introduction, the mental stability could be related to sympathetic reduction and improvement in the parasympathetic activity contributing to stress and anxiety reductions. Many other studies about the effects of meditation practice have been indicating a number of physiological changes, such as increased GABA concentration [57], adrenalin and noradrenalin reductions [58], increased serotonin [59] and melatonin concentrations [60], which can be related to improvements on mood, reductions in body temperature [61, 62] and increased galvanic skin resistance [63], may be related to stress management, and also increased gray matter density in brain regions related to emotional regulation [64]. New studies with familial caregivers would evaluate more physiological parameters than presented in this study. 

## 5. Conclusions

The results our study demonstrate are that the practice of yoga and the suggested meditation techniques may represent an effective intervention for caregivers of relatives with Alzheimer's disease to reduce their stress, anxiety, and depression symptoms and to decrease their cortisol levels. 

## Figures and Tables

**Table 1:** Frequency of the distribution of the phases of stress manifestation among the sampled individuals according to group before and after intervention.

Phases of stress manifestation	Before intervention	After intervention
	Control group	Control group
Absence	0 (0)	0 (0)
Alert	0 (0)	0 (0)
Resistance	18 (86)	16 (76)
Quasiexhaustion	3 (14)	4 (19)
Exhaustion	0 (0)	1 (5)

	YCMP group	YCMP group^&^
Absent	0 (0)	17 (68)*
Alert	0 (0)	0 (0)
Resistance	20 (80)	8 (32)*
Quasiexhaustion	3 (12)	0 (0)
Exhaustion	2 (8)	0 (0)

Data described as frequency (percentage); ^&^ *P* < 0.05: differs from control group, chi-square test; *P < 0.05: differs from time-point before intervention, *McNemar*'s test.

YCMP: yoga and compassion meditation program.

**Table 2:** Scores of depression and anxiety measured by BDI and BAI of familial caregivers of Alzheimer's disease patients before and after intervention.

	Before	After	*F*_time-point(1.44)_	*F*_group(1.44)_	*F*_inter(1.44)_
BDI					
Control group	14.7 (±9.5)	16.1 (±10.2)	16.5	2.0	34.7
YCMP group	16.0 (±7.8)	8.2 (±4.6)^∗&^	*P* < 0.001	*P* = 0.16	*P* < 0.000001
BAI					
Control group	19.9 (±10.2)	21.9 (±10.6)	12.9	9.6	32.3
YCMP group	17.6 (±9.4)	8.7 (±5.5)^∗&^	*P* < 0.001	*P* < 0.001	*P* < 0.00001

Data are described as the mean (±standard deviation); *P < 0.001 differs from the time point before the intervention in the corresponding group—*Tukey's *test; ^&^ *P* < 0.001 differs from the control group in the corresponding time point—*Tukey*'s test*.*

YCMP: yoga and compassion meditation program.

**Table 3:** Salivary cortisol concentration (ng/dL) of familial caregivers of Alzheimer's disease patients before and after intervention.

	Before	After	*F*_time-point(1.35)_	*F*_group(1.35)_	*F*_inter(1.35)_
Cortisol day 1					
Control group	539.4 (±152.6)	531.1 (±53.7)	3.64	0.10	3.31
YCMP group	743.9 (±140.7)	404.4 (±49.5)	*P* = 0.06	*P* = 0.76	*P* = 0.08

	Before	After	*F*_time-point(1.32)_	*F*_group(1.32)_	*F*_inter(1.32)_

Cortisol day 2					
Control group	674.7 (±181.7)	673.2 (±86.3)	7.42	0.00004	7.33
YCMP group	937.6 (±171.3)	408.1 (±81.3)*	*P* < 0.05	*P* = 0.99	*P* < 0.05

	Before	After	*F*_time-point(1.40)_	*F*_group(1.40)_	*F*_inter(1.40)_

Cortisol days 1 and 2					
Control group	641.5 (±137.6)	568.0 (±58.03)	8.38	0.02	3.97
YCMP group	823.0 (±131.2)	424.6 (±55.3)*	*P* < 0.001	*P* = 0.88	*P* < 0.05

Data are described as the mean (±standard deviation); *P < 0.05 differs from the time point before the intervention in the corresponding group—*Tukey's *test.

YCMP: yoga and compassion meditation program.
